# Modelling t(8;21) acute myeloid leukaemia ‐ What have we learned?

**DOI:** 10.1002/mco2.30

**Published:** 2020-09-24

**Authors:** Paulynn Suyin Chin, Constanze Bonifer

**Affiliations:** ^1^ Institute of Cancer and Genomic Sciences, College of Medical and Dental Sciences University of Birmingham Birmingham UK

**Keywords:** acute myeloid leukaemia, AML, patient‐derived xenograft, PDX, RUNX1‐ETO, t(8;21), transgenic mice

## Abstract

Acute myeloid leukaemia (AML) is a heterogeneous haematopoietic malignancy caused by recurrent mutations in haematopoietic stem and progenitor cells that affect both the epigenetic regulatory machinery and signalling molecules. The t(8;21) or RUNX1‐RUNX1T1 translocation generates the RUNX1‐ETO chimeric transcription factor which primes haematopoietic stem cells for further oncogenic mutational events that in their sum cause overt disease. Significant progress has been made in generating both *in vitro* and *in vivo* model systems to recapitulate t(8;21) AML which are crucial for the understanding of the biology of the disease and the development of effective treatment. This review provides a comprehensive overview of the *in vivo* and *in vitro* model systems that were developed to gain insights into the molecular mechanisms of RUNX1‐ETO oncogenic activity and their contribution to the advancement of knowledge in the t(8;21) AML field. Such models include transgenic mice, patient‐derived xenografts, RUNX1‐ETO transduced human progenitor cells, cell lines and human embryonic stem cell model systems, making the t(8;21) as one of the well‐characterized sub‐type of AML at the molecular level.

## Graphical abstract

The t(8;21) or RUNX1‐RUNX1T1 translocation generates the RUNX1/ETO chimeric transcription factor which in cooperation with other mutations drives one of the most frequent types of acute myeloid leukaemia. Here, we provide a comprehensive overview of the *in vivo* and *in vitro* model systems that informed about the molecular mechanisms of RUNX1/ETO oncogenic activity.

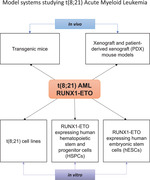

## 1 INTRODUCTION

Acute myeloid leukaemia (AML) is characterised by uncontrolled proliferation of myeloid progenitor cells with impaired myeloid differentiation which results in an accumulation of immature cells in the bone marrow that rapidly interfere with the generation of normal blood cells. AML is one of the most common leukaemia in adults which accounts for 25% of all adult leukaemia. The average age of AML patients is 65+ years and older patients are predisposed to higher incidence of relapse.^1^

**FIGURE 1:**
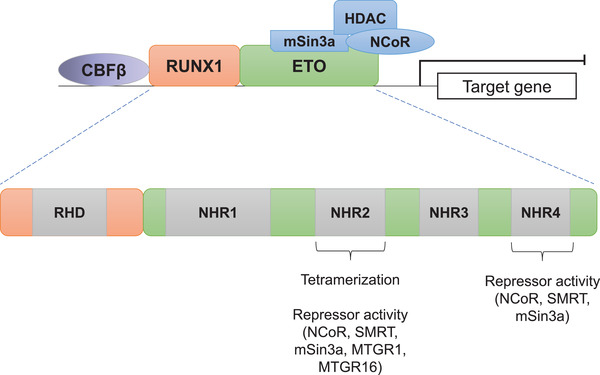
Structure of the RUNX1‐ETO fusion protein and interaction partners. The fusion of RUNX1 with ETO protein results in a formation of a transcriptional repressor via the recruitment of the mSin3a/NCoR/HDAC complex. The runt homology domain (RHD) of RUNX1 which contains the DNA binding domain is retained. The ETO protein interacts with the repressor complex via the *nervy homology domains* (NHR) 2 and 4

AML develops as a result of recurrent mutations in haematopoietic stem and progenitor cells (HSPCs) that affect both the gene regulatory machinery and molecules involved in multiple signalling pathways, such as transcription factors/chromatin modifiers and growth factor receptors, respectively. AML consists of a variety of subtypes with different clinical characteristics and the t(8;21) AML is one of the best‐studied subtypes at the molecular level. In this translocation, the *RUNX1* (also referred to as *AML1*) gene on chromosome 21 is fused to almost the entire *ETO* (also referred to as *RUNX1T1* or *MTG8*) gene on chromosome 8. The translocation product still contains the DNA binding domain of RUNX1 but the activation domain of RUNX1 is replaced by ETO and thus, the RUNX1‐ETO (also referred to as RUNX1‐RUNX1T1 or AML1‐ETO) fusion acts as a transcriptional repressor. Extensive structural and functional studies aimed at understanding the role of the different domains of the RUNX1‐ETO fusion protein and its interaction partners (Figure 1) shed light on its function in the genome.^2^, ^3^, ^4^ It was shown that ETO forms a stable repressor complex with the nuclear receptor corepressor (NCoR), the mSin3A corepressor and recruits histone deacetylases. These interactions are mediated by the four conserved *nervy homology regions* (NHR1‐4) of the ETO protein. NHR1 interacts with E proteins, whereas NHR2 forms a tetramer which is critical for RUNX1‐ETO leukaemogenic potential.^5^, ^6^, ^7^ NHR2 mediates oligomerization of RUNX1‐ETO, ETO, or the other ETO family members MTGR1 and MTGR16.^6^ The contribution of NHR3 to RUNX1‐ETO function is less well characterised but it was found that it interacts with the regulatory subunit of type II cyclic AMP‐dependent protein kinase.^8^ Similar to NHR2, NHR4 also plays an important role in leukaemogenesis and it binds to NCoR complexes and the silencing mediator of retinoid and thyroid hormone receptor (SMRT).^2^, ^9^

**FIGURE 2:**
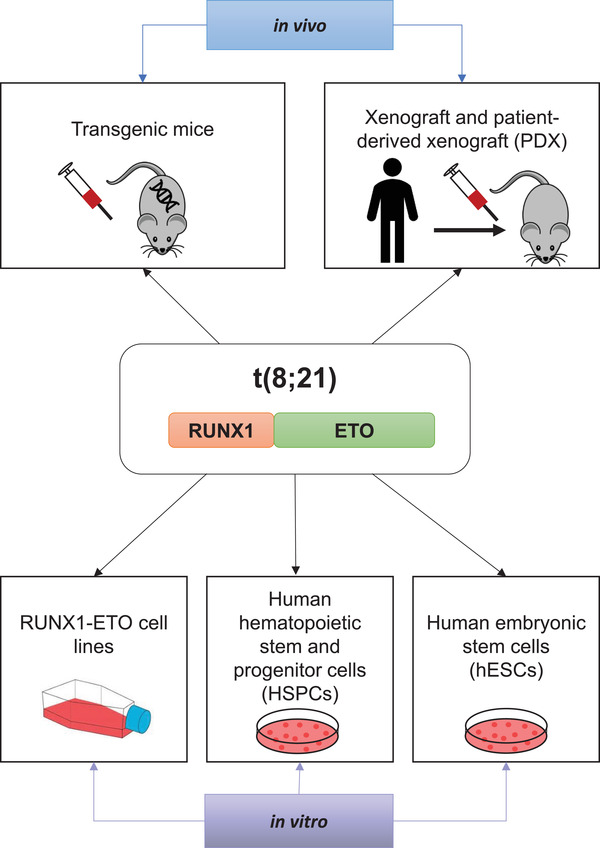
Summary of the in vivo and in vitro model systems that were developed to gain insights into the global mechanisms of RUNX1‐ETO fusion protein action

Although t(8;21) AML patients have a relatively favourable prognosis, this is not the case for elderly patients who are unable to tolerate intensive chemotherapy and many t(8;21) patients will relapse after initial remission. Therefore, new drugs or new personalised therapies are needed. Since drug development cannot start with patients, model systems that recapitulate aspects of specific AML sub‐types are crucial for the understanding and development of effective treatments. This review provides a comprehensive overview of the in vivo and in vitro model systems that were developed (Figure 2) to gain insights into the global mechanisms of RUNX1‐ETO fusion, their advantages and disadvantages and what we have learned from such experiments.

## 2 IN VIVO MODELS

### 2.1 Transgenic models

In order to validate the function of RUNX1‐ETO during AML initiation and disease progression, many studies have used mouse models which led to novel discoveries of target genes and treatment therapies in the AML field. One of the earliest RUNX1‐ETO mice models involved a RUNX1‐ETO fusion gene knock‐in into the murine RUNX1 locus, generating heterozygous embryos which led to embryonic lethality due to the absence of definitive foetal liver‐derived haematopoiesis and lethal haemorrhages.^10^ This phenotype was similar to the homozygous RUNX1 knockout mice.^11^ However, the phenotype was not identical because yolk sac cells and foetal liver cells from the RUNX1‐ETO knock‐in embryos were able to generate macrophages and dysplastic multilineage haematopoietic progenitors, respectively, when cultured *in vitro*.^10^, ^12^ These studies showed that RUNX1‐ETO not only has a dominant negative effect on the wild‐type activity of RUNX1 but can induce leukaemogenesis.

To overcome the embryonic lethality phenotype of the RUNX1‐ETO knock‐in model, other studies have moved towards generating conditional transgenic models where RUNX1‐ETO can be conditionally expressed in adult bone marrow progenitors. This was achieved via CRE/LoxP‐mediated recombination where RUNX1‐ETO fusion was induced only in the presence of Cre recombinase.^13^, ^14^ Subsequently, mice expressing RUNX1‐ETO under the control of the tetracycline (Tet) promoter were generated.^15^ Interestingly, in both systems, no leukaemia developed, although mice expressed high levels of RUNX1‐ETO in the bone marrow and progenitors showed abnormal maturation and increased self‐renewal. Similarly, mice expressing RUNX1‐ETO under the control of the hMRP8 promoter failed to develop leukaemia as well.^16^ However, upon exposure to a strong carcinogenic mutagen, N‐ethyl‐N‐nitrosourea, these mice developed a full blown AML, suggesting that additional mutations were necessary for transformation.^14^, ^16^ Subsequently, another inducible mouse model of RUNX1‐ETO under the control of ROSA26 locus which allows for conditional and reversible controlled mosaic expression of RUNX1‐ETO was developed.^17^ Transcriptomic analysis from different haematopoietic stem cell (HSC) populations showed that the transcriptional changes in malignant cells recapitulated what was observed in human t(8;21) AML. Long‐term overexpression of RUNX1‐ETO in mice also led to a myeloproliferative disease (MPD)‐like myeloid leukaemia phenotype with a high white blood cell count and with increased immature bone marrow granulocytes and circulating blasts in the periphery. However, all mice survived. Subsequent studies have then focused on generating mosaic models where RUNX1‐ETO was targeted into the HSCs compartment using retroviral transduction and these cells were then transplanted into lethally irradiated mice. The transplanted mice expressing RUNX1‐ETO in HSCs recapitulated the haematopoietic developmental abnormalities observed in patients with the t(8;21) translocation such as abnormal basophilic granulation, but again without developing AML.^18^ Taken together, these studies showed that RUNX1‐ETO alone is unable to cause leukaemia and additional mutations are required for leukaemogenesis.

To elucidate the nature of the cooperating mutations, later studies have therefore focused on the cooperation of RUNX1‐ETO with other mutations such as deficiency of interferon regulatory factor ICSBP^19^ or activating mutation in NRAS and tyrosine kinases such as *c‐KIT*, *Flt3*, and *TEL‐PDGFβR*.^20^, ^21^, ^22^, ^23^, ^24^ To study the cooperation of the tumour suppressor of myeloid neoplasia, ICSBP with RUNX1‐ETO, bone marrow cells derived from ICSBP deficient mice were transduced with RUNX1‐ETO.^19^ The cooperation between RUNX1‐ETO and ICSBP deficiency resulted in the induction of myeloid tumorigenesis and the development of non‐fatal granulocytic sarcomas. Because activating mutations in receptor tyrosine kinases are present in at least 30% of patients with AML, multiple studies investigated their cooperation with RUNX1‐ETO by retrovirally transducing murine bone marrow cells with RUNX1‐ETO and either the *TEL‐PDGFβR* fusion protein, the *c‐KIT* N822K and D814V mutations, the *Flt3* length mutation (FLT3‐LM) or NRas^G12D^ mutation into lethally irradiated syngeneic mice.^20^, ^21^, ^22^, ^23^, ^24^, ^25^ In all studies, RUNX1‐ETO cooperated with these activating mutations and caused AML. However, using retroviral transduction to over‐express cooperating mutations can lead to expression of the respective proteins at non‐physiological levels within the cells and thus, may not precisely recapitulate the effect of the mutations on HSC function.^26^ To address this issue, a study was conducted using a conditional knock‐in of RUNX1‐ETO and KRas^G12D^ into Mx1‐Cre mice HSC, where they are expressed from their own promoter at their original loci to reproduce physiological oncogene expression levels within the haematopoietic hierarchy.^27^ Interestingly, co‐expression of RUNX1‐ETO and KRas^G12D^ resulted in a detrimental effect on HSCs with loss of quiescence and self‐renewal‐associated gene expression. Contradicting to previous studies, this cooperation therefore was insufficient to cause transformation of myeloid progenitors. The study also explained why oncogenic RAS mutations always occur as secondary mutations and are never found in HSCs.

Besides cooperation with signal transduction mutations, RUNX1‐ETO was also found to cooperate with the WT1 transcription factor with the expression of both rapidly causing leukaemia.^28^ Mice overexpressing both WT1 and RUNX1‐ETO in haematopoietic cells displayed inhibition of myeloid differentiation at more immature stages as compared to cells transduced with RUNX1‐ETO alone. Moreover, co‐transplanted mice rapidly developed AML. Therefore, this study suggests that WT1 may play an oncogenic role by altering proliferation and differentiation of haematopoietic cells.

It has been reported that a version of RUNX1‐ETO truncated at the carboxy‐terminal end of the protein (RUNX1‐ETO9a) was able to induce AML in murine HSPCs on its own.^29^, ^30^, ^31^ Expression of RUNX1‐ETO9a led to rapid induction of leukaemia in mice and co‐expression of RUNX1‐ETO and RUNX1‐ETO9a resulted in an earlier onset of AML with a block in myeloid differentiation at a more immature stage.^29^ High expression of RUNX1‐ETO9a was detected in a proportion of (86 out of 118) paediatric and adult t(8;21) AML which correlated with poor clinical outcome and increased c‐KIT expression.^31^ However, a subsequent study showed that HSPCs expressing RUNX1‐ETO and RUNX1‐ETO9a at physiological levels were unable to develop leukaemia.^32^ Similarly, conditional RUNX1‐ETO9a knock‐in mice showed enhanced proliferation and re‐plating capacity but did not develop AML.^33^ Additionally, contrasting to previous findings, the 9a protein was not detectable in all t(8;21) patient samples.^32^ More recently, using a more sensitive RT‐PCR methodology, RUNX1‐ETO9a was detected in all 129 t(8;21) AML patients,^34^ but expression of this isoform did not correlate with any clinical feature, throwing the clinical relevance of the original finding into question.

### 2.2 Xenograft and patient‐derived xenograft models

Whilst transgenic mouse models have contributed significantly to the AML field, it is becoming increasingly clear that there are fundamental discrepancies between mouse models and the human system, with the multistep tumour progression in mice being much less complex as compared to what is observed in humans. In addition, differences in telomere length as well as differences in RAS signalling between the two species have been observed.^35^, ^36^ Therefore, the impact of RUNX1‐ETO expression in mouse haematopoietic cells may be different compared to human haematopoietic cells. Expansion of human AML cells *in vitro* has also proved to be technically challenging as culture systems do not mimic the complex interaction between the leukaemic cells and the bone marrow microenvironment. Therefore, xenograft models have been developed where AML cell lines or patient‐derived AML blast cells are engrafted into immunocompromised mice.

The earliest attempts to transplant AML cell lines or primary AML cells were conducted on athymic nude mice that were homozygous for the nude (*Foxn1*) gene which lack functional T cells.^37^ However, engraftment was poor due to the presence of B and NK cells which provoked the thymus‐independent immune system. In addition, the engrafted mice generated granulocytic sarcomas without bone marrow engraftment. To overcome these problems, severe combine immunodeficient (SCID) mouse models were created. These mice harbour inactivating mutations in the *Prkdc* gene and thus lack functional T or B cells but retain NK functions. Although primary AML cells were principally capable of engrafting and proliferating in SCID mice and retained their biological and clinical behaviour observed in human AML, engraftment rates remained poor.^38^ This problem was caused by the lack of species cross‐reactivity of cytokines and an innate host resistance because of elevated NK cell function. To improve engraftment rates, supplementation with human cytokines and growth factors including stem cell factor, granulocyte‐macrophage‐colony stimulating factor, IL‐3, human mast cell growth factor and erythropoietin resulted in enhanced engraftment of human cells.^39^ However, the elevated NK cells function leading to host resistance remained as a problem affecting the engraftment efficiency.^40^

Further work to improve this model has led to the development of a non‐diabetic (NOD/SCID) mouse model lacking functional B and T cells and displaying reduced NK cell and macrophage activity.^41^ This alteration significantly improved engraftment rates, requiring fewer human AML cells and for the first time, CD34^+^ CD38^−^ SCID leukaemia‐initiating cells (SL‐IC) could be identified which had the potential for self‐renewal and could repopulate immune‐deficient mice and were subsequently named leukaemic stem cells.^42^ Interestingly, a later study showed that also the CD34^+^ CD38^+^ fraction can repopulate in NOD/SCID mice.^43^ In terms of RUNX1‐ETO, CD34^+^ cord blood cells expressing RUNX1‐ETO showed good engraftment in NOD/SCID mice. However, even after a prolonged observation period, none of the mice developed AML,^44^ again demonstrating that additional mutations are required for leukaemogenesis.

Subsequently, a further immunocompromised NOD/SCID mice model was developed by deletion of the gamma chain of IL‐2R (NOD/SCID/IL2rγ^−/−^, NSG).^45^ This deletion resulted in an almost complete loss of murine immune system and thus, significantly improved AML engraftment.^46^ A study comparing the engraftment kinetics of AML and ALL cell lines including the RUNX1‐ETO Kasumi‐1 cells in the NOD/SCID and NSG models has shown that Kasumi‐1 successfully engrafted in NSG but not in NOD/SCID mice.^47^ Although engraftment of t(8;21) patient cells remained challenging, engraftment of human RUNX1‐ETO cell lines in NSG models are commonly used for therapeutic treatment studies. Treatment with a small molecule, named Compound 7.44 that disrupts RUNX1‐ETO tetramerization in NSG mice engrafted with Kasumi‐1 cells, resulted in reduced spreading of leukaemic cells and prolonged survival in the mice.^48^ In order to find crucial RUNX1‐ETO responsive target genes, NSG mice transplanted with either Kasumi‐1 or SKNO‐1 cells were transduced with a RNAi library that targeted RUNX1‐ETO target genes.^49^ This has led to the identification of the *CCND2* gene as being essential for leukaemic maintenance and self‐renewal*in vivo*. Furthermore, using Rag2^−/−^ IL2rg^−/‐^ 129xBalb/c mice engrafted with Kasumi‐1 cells, the same study showed that inhibition of CCND2 by treatment with palbociclib (CDK4/6 inhibitor) inhibited leukaemia development with prolonged survival of transplanted mice.

A more recent and further improved mouse model, MISTRG, was created by knocking in a mix of human cytokines into the endogenous mouse loci, creating a microenvironment that supported human cells even better.^50^ The presence of human cytokines in MISTRG mice supported the development of functional human myeloid cells, including monocytes, macrophages and NK cells from human foetal liver or adult CD34^+^ cells injected into the mice. Although there is still no evidence for the engraftment of t(8;21) in these mice, it has been shown that inv(16) AML, which is also a core binding factor (CBF) leukaemia could successfully engraft.^51^ Therefore, it would be interesting to determine whether the MISTRG can improve the engraftment of t(8;21) AML cells which have been very challenging in the other model systems.

It has been shown that the Bcl‐xL pathway as well as the PI3K/AKT/mTOR pathway mediated by thrombopoietin/MPL (THPO/MPL) plays a critical role in self‐renewing and maintenance of RUNX1‐ETO cells.^52^, ^53^ In addition, upregulation of MPL expression cooperates with RUNX1‐ETO to cause leukaemia in mice. Although MPL expression was detected in all t(8;21) AML, only a proportion of the t(8;21) cells expressed high MPL protein levels. Therefore, preferentially selecting for cells with high‐MPL expression to engraft in MISTRG mice that has a knock‐in THPO cytokine might improve the t(8;21) engraftment.

## 3 IN VITRO MODELS

### 3.1 RUNX1‐ETO cell lines

Although *in vivo* models have given us great insights into the cell biology of AML development and provided us with the means for drug testing in an appropriate context, the actual drug development requires to perform high‐throughput assays with a large number of cells. To this end, the human Kasumi‐1 and SKNO‐1 cell lines are a widely used and well‐characterised model system for t(8;21) AML.^54^, ^55^, ^56^ The Kasumi‐1 cell line was established from the peripheral blood of an AML patient after relapse and bone marrow transplantation, whereas the SKNO‐1 cell line was derived from the bone marrow cells of an AML patient whose disease became resistant to chemotherapy.^54^, ^56^ These cell line models have allowed us to gain important insights into the molecular mechanisms mediating RUNX1‐ETO oncogenic activity. Depleting RUNX1‐ETO using siRNA in these cells led to upregulation of genes associated to myeloid differentiation and thus, indicating that continued expression of RUNX1‐ETO is important in the maintenance of t(8;21) AML.^55^ Subsequent studies showed that a significant proportion of genes upregulated by RUNX1‐ETO depletion had increased RNA Polymerase II enrichment and a genome‐wide increase in H3K9 acetylation as well as a redistribution in RUNX1 binding.^57^ Similarly, a follow‐on study using the same system in Kasumi‐1 cells showed a dynamic equilibrium between RUNX1 and RUNX1‐ETO binding and that binding to their target sites is mutually exclusive.^58^

Proteomics experiments using Kasumi‐1 cells showed that RUNX1‐ETO does not act alone, but is part of a larger transcription factor complex with the ETS family of transcription factors (ERG and FLI1); E proteins such as HEB, E2A, and LYL1; and the non‐DNA‐binding factors LDB1 and LMO2.^59^, ^60^ More recently, by using the Promoter Capture Hi‐C method on Kasumi‐1 cells with and without RUNX1‐ETO depletion, it was shown that RUNX1‐ETO depletion affects the chromatin architecture within the nucleus, which is driven by increased binding of the myeloid transcription factors, C/EBPα, driving cell differentiation, and loss of AP‐1 binding.^61^ siRNA‐mediated RUNX1‐ETO depletion usually takes at least 2 days, but transcriptional regulatory networks can respond within minutes to hours, making it difficult to distinguish the direct targets from secondary or compensatory transcriptional changes. Therefore, an elegant recent approach was developed using CRISPR technology to insert a degron tag into the endogenous RUNX1‐ETO locus of Kasumi‐1 cells. The addition of a small molecule led to rapid degradation of RUNX1‐ETO protein by proteolysis within the first 30 minutes to 2 hours.^62^ This study identified a RUNX1‐ETO‐regulated core circuit consisting of 60 genes, including genes associated to myeloid differentiation and cell fate decisions.

Although studies in mice suggest that RUNX1‐ETO has a dominant negative effect on wild‐type RUNX1, further evidence suggests that a certain level of wild‐type RUNX1 is crucial for leukaemogenesis.^63^, ^64^ Knockdown of RUNX1 in Kasumi‐1 cells led to apoptosis, which could be rescued by knockdown of RUNX1‐ETO. Taken together, these data suggest that a balance of wild‐type RUNX1 and RUNX1‐ETO is important to sustain the malignant cell phenotype of t(8;21) AML.

### 3.2 Human haematopoietic stem and progenitor cells

Cell lines are adapted to cell culture conditions and have therefore lost many of the characteristics of primary cells. Many studies have therefore focused on introducing RUNX1‐ETO by retroviral or lentiviral delivery systems into primary human HSPCs.^65^, ^66^, ^67^, ^68^, ^69^, ^70^, ^71^ Both the CD34^+^ bone marrow or mobilized peripheral blood stem cells (PBSCs) and CD34^+^ cord blood cells were used for transduction but in the field there is a preference for CD34^+^ cord blood cells as these cells were found to engraft better in NOD/SCID mice and can be more easily obtained.^72^ We have recently shown that both CD34^+^ cord blood cells and CD34^+^ PBSCs are very similar in terms of open chromatin regions and are enriched for genes specific for the different myeloid progenitor cell types at a very similar level (Chin et al., submitted).

RUNX1‐ETO expressing CD34^+^ cord blood cells maintain self‐renewal and multipotent differentiation, but when transplanted do not cause leukaemia in NOD/SCID mice,^65^ consistent with the transgenic mouse models. However, and in contrast to the mouse models, the introduction of additional mutant oncoproteins, in this case KIT (N822K), NRAS (G12D) or mutant CBL into RUNX1‐ETO CD34^+^ PBSCs and CD34^+^ cord blood cells, still failed to initiate AML in immunodeficient mice, suggesting that even more additional cooperating factors are required or that cells need to undergo a strong selection in culture.^73^, ^74^, ^75^ However, transduction of RUNX1‐ETO with the telomerase enzyme hTERT immortalised RUNX1‐ETO CD34^+^ HSPCs and resulted in full transformation which recapitulated the disease progression in patients. This behaviour is likely to be due to the unlimited proliferative potential mediated by hTERT and allowing for the acquisition of additional mutations to cause an overt disease in AML.^69^

In addition, over‐expressing RUNX1‐ETO in HSPCs has allowed to robustly produce large numbers of transformed cells enabling global analyses of RUNX1‐ETO effects on the epigenome in a human setting. We have shown that FOXO1 plays a role in AML leukaemogenesis as it is required for the activation of the self‐renewal program in RUNX1‐ETO CD34^+^ cord blood cells and thus presents a potential therapeutic target.^68^ More recently, our group compared the open chromatin profile from *in vitro*‐generated CD34^+^ cord blood cells co‐expressing both RUNX1‐ETO and c‐KIT (N822K) to that of t(8;21) patients and found that although both display the AP‐1 motif signature, open chromatin regions in patient cells displayed an increased inflammatory signature in the form of enriched NF‐κB motifs, which was not observed in the *in vitro*‐generated cells (Chin et al., submitted). We speculate that this could be due to the fact that in vitro cells are grown in culture conditions that is different from the bone marrow niche and thus, shape the chromatin landscape in a different way. Degradation experiments using the degron tag approach discussed earlier in primary RUNX1‐ETO expressing CD34^+^ cord blood cells compared RUNX1‐ETO responsive genes in these cells with those identified in Kasumi‐1 cells, identified many overlapping genes but also found a number of differences.^62^

### 3.3 Human embryonic stem cells

The role of RUNX1‐ETO has also been investigated in human myeloid‐differentiated‐induced pluripotent stem cells (iPSCs).^76^ Consistent with the other models, RUNX1‐ETO induces leukaemic characteristics and overexpression led to increased apoptosis. Subsequently, to study the effects of RUNX1‐ETO itself in an early stage, an inducible human iPSCs model that allows*in vitro* differentiation towards different mature myeloid cell types with inducible expression of RUNX1‐ETO at the different stages was developed.^77^ This system has enabled global transcriptome and epigenome analysis and demonstrated that RUNX1‐ETO, in the absence of additional mutations, alters the myeloid gene program by altering the acetylome of differentiating granulocytic cells and increased self‐renewal capacity. However, the cells generated by this iPSC differentiation system resembled yolk‐sac‐derived progenitors and were unlikely to represent the proper target cell types. Therefore, we generated cells allowing inducible expression of RUNX1‐ETO in a human embryonic stem cell (hESC) differentiation system that produced definitive multipotent haematopoietic progenitor cells.^78^ This strategy allowed us to study the initial reprogramming events imposed on the myeloid gene regulatory network by RUNX1‐ETO. RUNX1‐ETO induction led to a rapid reprogramming of the epigenome by interference with RUNX1 binding, the induction of quiescence, and the promotion of cell survival. Moreover, using single‐cell approaches, we were for the first time able to identify the actual target cell type responding to RUNX1‐ETO expression showing it to be an early myeloid progenitor cell. In contrast to other progenitor types, such as erythroid progenitors or cells further along the myeloid differentiation pathway, these cells downregulated expression of the myeloid master regulators PU.1 and C/EBPα and showed a G1 cell cycle block, thus halting differentiation. Taken together, this hESC differentiation system is a promising *in vitro* system for future studies of RUNX1‐ETO function and can be used to study multiple different oncogene types.

## 4 CONCLUSIONS

Despite improvement in t(8;21) model systems, robust expansion of t(8;21) AML blasts *in vitro* while maintaining their naïve characteristics and improvement in engraftment of these cells in mice remains a challenging task. Unfortunately, no specific current model can faithfully recapitulate the complexities of the AML human disease which warrants ongoing efforts in developing a next generation mouse model suitable for system‐wide studies and drug testing. A promising new development is patient‐derived xenograft models carrying subcutaneous humanized bone marrow ossicles thus providing humanized niche.^79^, ^80^ Although the t(8;21) AML cells did not engraft, other primary AML cells showed much improved levels of engraftment as compared to NSG mice. This model could therefore be further improved for successful t(8;21) engraftment. Such improvements will eventually lead to a better understanding of the molecular pathogenesis of t(8;21) AML and allow development of novel targeted and personalised treatment therapies.

## CONFLICT OF INTEREST

The authors declare no conflict of interest.

## AUTHOR CONTRIBUTIONS

PSC and CB wrote the paper.
